# Cellular uptake, transport mechanism and anti-inflammatory effect of cyanidin-3-glucoside nanoliposomes in Caco-2/RAW 264.7 co-culture model

**DOI:** 10.3389/fnut.2022.995391

**Published:** 2022-09-26

**Authors:** Mengyu Yang, Xiaoqin Lu, Jie Xu, Xiaofeng Liu, Wei Zhang, Rongfa Guan, Hao Zhong

**Affiliations:** College of Food Science and Technology, Zhejiang University of Technology, Hangzhou, China

**Keywords:** cyanidin-3-glucoside, uptake, transport, anti-inflammation, co-culture

## Abstract

Cyanidin-3-glucoside (C3G), which is the widest and richest anthocyanin (ACN) found in the edible fruit and vegetables, has been illustrated to perform a wide range of bioactivities. Nanoliposomes can inhibit C3G degradation and enhance the absorption rate of C3G as tools for conveying materials to particular locations. This experiment aims to study the absorption, transport and anti-inflammatory effects of C3G nanoliposomes in Caco-2/RAW 264.7 co-culture model, which symbolizes an intestinal inflammation system. The results indicated that the uptake and transport of C3G nanoliposomes by Caco-2/RAW 264.7 co-culture model were concentration-dependent as well as affected by temperature (37 and 4°C) and endocytic inhibitors, which revealed C3G nanoliposomes penetrate cells *via* endocytosis. Moreover, compared with C3G, C3G nanoliposomes significantly decreased pro-inflammatory cytokine expression (tumor necrosis factor (TNF)-α, interleukin (IL)-1β, IL-6, IL-8), suggesting a stronger anti-inflammatory potential. Conclusively, the uptake of C3G nanoliposomes by Caco-2/RAW 264.7 co-culture model is mainly involved in macropinocytosis and endocytosis mediated by carrier protein (clathrin). C3G nanoliposomes may play a better role in the treatment of LPS-induced intestinal inflammation diseases.

## Introduction

Cyanidin-3-O-glucoside (C3G) is the most abundant anthocyanin in plant-based foods such as black rice, purple potatoes, blackberries, black grapes, blood oranges, mulberries and other colored grains, fruits and vegetables (1). Owing to fresh colors, abundant reserves, and various bioactivities, C3G has become a prevalent research object in the field of flavonoids relatively. Recent research has reported that C3G has anti-inflammatory, antioxidant, anti-proliferation, anti-cancer, neuroprotective and anti-diabetic activities (2). However, the absorption efficiency and efficacy of C3G are limited in the human body due to its low stability and bioavailability. Thus, strategies to improve bioavailability and controlled release rate are of great importance for exploring absorption mechanisms and other functional applications of C3G.

Nanoliposomes are nanoscale liposomes that are composed of phospholipids with hydrophobic and hydrophilic phases (3). Phospholipids, as the major component can encapsulate bioactive substances to enhance bioavailability and help bioactive materials transport across the cell membranes with fluidity. As C3G has several hydroxide radicals and flavylium with charge, they could combine with the hydrophilic phase of liposome (4). C3G nanoliposome (C3G-NL) could replace phospholipids by incorporating them with the cell membrane to improve the transport efficiency and uptake rate of C3G molecule (5). Therefore, not only can encapsulated materials be delivered to the specific target site for treatments, but reduce the C3G leakage rate and improve its stability.

Many single bioactive substances, extracts of functional food, and several drugs were conveyed to the human body *via* gastrointestinal absorption (1). Moreover, the intestinal barrier and the integrity of intestinal epithelial monolayer are crucial to keep host homeostasis because they could inhibit luminal antigens invasion (6). An “intestinal leakage,” which signifies the damage of the intestinal epithelium's protective layers would promote intestinal inflammation's development (7). This may lead to the overproduction of oxide and peroxide, the release of inflammatory cytokines from gut and immune cells, resulting in severe continuous mucous membrane injury and improvement of infection (8). As a result, the relationship between the penetrated bowel and inflammatory response remains bilateral and relative.

In this research, the characterization of the C3G and C3G-NL was illustrated through transmission electron microscopy (TEM), dynamic light scattering (DLS), fourier transform infrared spectroscopy (FTIR). Caco-2 cells (human clonal colonic adenocarcinoma cells) were incubated on the apical (AP) side of a tissue culture plate insert. RAW 264.7 cells (mononuclear macrophages in mice) were incubated on the basolateral (BL) side to interact with Caco-2 cells *via* indirect contact. The stability and uptake of C3G from the nanoliposomes were compared to those of C3G molecules using the Caco-2/RAW 264.7 co-culture system. The anti-inflammatory properties of the C3G were measured through inflammatory cytokines such as TNF-α, IL-1β, IL-6, IL-8.

## Materials and methods

### Chemicals

Caco-2 cells, fetal bovine serum (FBS), minimum essential medium (MEM) glucose medium and Dulbecco's modified eagle medium (DMEM) were bought from Shanghai QIDA Biological Science and Technology Ltd (Shanghai, China). RAW264.7 cells were obtained from Hunan FENGHUI Biological Science and Technology Ltd (Changsha, China). Phosphate buffer solution (PBS), penicillin and streptomycin, HEPES (1 M) buffer, glutamax, non-essential amino acids (NEAA) and 0.25% trypsin-EDTA were obtained from Beyotime (Shanghai, China). Cyanidin-3-glucoside (C3G) was bought from Chengdu Biopurify Phytochemicals Ltd (Chengdu, China). Tissue culture plate inserts (6.5 mm membrane diameter, 0.4 μm aperture diameter) were obtained from Hangzhou NUOYANG Biological Science and Technology Ltd (Hangzhou, China). Cell Counting Kit-8 (CCK-8 Kit) and Alkaline Phosphatase Kit was bought from Nan Jing Jian Cheng Bioengineering Institute (Nanjing, China). Lucifer yellow (LY) was purchased from Solarbio (Beijing, China). Phosphatidylcholine (PC), cholesterol (CH), and lipopolysaccharide (LPS) were purchased from Shanghai YUANYE Biological Science and Technology Ltd (Shanghai, China). High-performance liquid chromatography (HPLC)-grade solvents, including methanol, acetonitrile, and phosphoric acid, were bought from Shanghai Macklin Biochemical Technology Co., Ltd (Shanghai, China). Coumarin-6, DAPI, methyl-β-cyclodextrin (β-CD), chlorpromazine (CPZ), cytochalasin-D (CD), phosphotungstic acid, potassium bromide (KBr) were bought from Sinopharm Chemical Reagent Co. LTD (Shanghai, China). TNF-α, IL-1β, IL-6, IL-8 ELISA kit were purchased from Wuhan Genmei Biotechnology Co., LTD (Wuhan, China).

### Preparation of C3G-loaded nanoliposomes

Nanoliposome preparation was made using the film dispersion method with some modifications (9). Briefly, 90 mg of phosphatidylcholine and 30 mg of cholesterol were dissolved in 20 mL of absolute ethanol. Meantime, 2 mg of C3G was dissolved in 10 mL of PBS as a sample solution (pH 6.8). Then 1 mL of sample solution was rapidly injected into the above organic solvent. The turbid liquid was stirred continuously by a magnetic agitator for 40 min until the solution became milky (water-in-oil emulsion). Subsequently, the ethanol in the emulsion was volatilized by the rotary evaporator under 41°C. Ultimately, C3G nanoliposomes (C3G-NL) were eluted by deionized water with ultrasonication. Blank nanoliposomes (B-NL, without C3G) were designed under the same condition except the C3G addition procedure.

### Measurement of C3G-NL encapsulation efficiency

The encapsulation efficiency (EE) of the C3G-NL was measured *via* centrifugal-ultraviolet spectrophotometry for a differential concentration between C3G and C3G-NL described previously with minor modifications (10). C3G in nanoliposomes were separated from liposomal system by a freeze centrifuge after charging absolute ethyl alcohol. Total C3G after demulsification was centrifuged at 8,000 rpm for 30 min under 4°C, and the total amount of C3G was detected at a wavelength of 531 nm by an ultraviolet spectrophotometer. The C3G-NL was centrifuged under the same conditions directly, the absorbance of free-C3G could be determined at the same wavelength at 531 nm. The EE percentage and leakage rate (LR) were determined by Equations (1) and (2), respectively.


(1) $$\begin{array}{l} EE\left(\%\right)=\frac{C_{2}-C_{1}}{C_{2}}\times 100\% \end{array}$$

(2) $$\begin{array}{l} LR\left(\%\right)=\left(1-\frac{EE_{t}}{EE_{0}}\right)\times 100\% \end{array}$$

where *C*_1_ represents the amount of free-C3G, and *C*_2_ represents the total C3G after demulsification of liposomes. EE_t_ is the encapsulation efficiency at time (*t*), and EE_0_ is the encapsulation efficiency of liposomes prepared initially.

### Determination of particle size and zeta-potential

The nanoliposome solution was diluted 10-fold before detected by a Brookhaven Particle Size and Zeta-Potential Analyzer. The device was operated by dynamic light scattering (DLS) to calculate and analyse the mean particle size, zeta-potential, polydispersity index (PDI) and the size of particles with diameters of 0.2–8,000 nm directly.

### The morphological structures of nanoliposomes

The morphological differences between B-NL and C3G-NL were determined by transmission electron microscopy (TEM). The sample was diluted 10-fold before being located on a copper grid, which was negative-stained *via* phosphotungstic acid solution for 2 min after aspiration of any excess liquid. The nanoliposomes-loaded copper grids were air-dried under infrared drying light and then observed under TEM.

### Fourier transform infrared (FTIR) spectroscopy

The C3G powder, freeze-dried B-NL and lyophilize C3G-NL were mixed with a proper amount of KBr. Each sample was recorded using FTIR spectrometer with the infrared spectra from 4,000 to 400 cm^−1^ wavenumber at a resolution of 4 cm^−1^.

### Cell culture

Caco-2 cells were incubated in MEM supplemented with 20% FBS, 1% NEAA, 1% L-glutamine and 1% penicillin/streptomycin at 37°C in a humidified incubator with 5% CO_2_. RAW 264.7 cells were maintained in DMEM containing 10% FBS, 1% sodium pyruvate, 1% penicillin/streptomycin at 37°C and 5% CO_2_.

### Cytotoxicity study

Following previously published literature, the cytotoxicity studies were assessed with a CCK-8 assay kit (11). The Caco-2 cells and RAW264.7 cells were respectively seeded into 96-well transwell plate at density of 1.0 × 10^5^ cells mL^−1^. After 24 h, Caco-2 cells were incubated with different concentrations of C3G and C3G-NL (0, 0.1, 0.2, 0.3, and 0.4 mg/mL) for another 24 h. RAW264.7 cells were incubated with different concentrations of LPS (0, 0.5, 1, 1.5, 2, and 2.5 μg/mL) and incubated for 24 h. Then, both types of cells were washed 3 times by PBS. 10 μL of the CCK-8 solution was added into each well and incubated for 4 h. Cell viability was measured using a microplate reader (ThermoFisher, USA) at a wavelength of 450 nm.

### Establishment of intestinal inflammation system

Caco-2 cells were grown in the AP side of a 24-Transwell plate at a density of 1 × 10^4^ cells/well for about 21 days. Before Caco-2 cells were differentiated completely, RAW 264.7 cells were seeded at a density of 1 × 10^5^ cells/well at a 24-well plate for 3 days. On 21st day, the AP side with polycarbonate (PC) film where Caco-2 cells had been seeded were transferred to the 24-well plate incubated with the RAW 264.7 cells as the BL side. The culture medium of both sides was changed with 10% FBS DMEM. To stimulate the production of inflammatory factors, LPS (1 μg/mL), as detected non-cytotoxic *via* the CCK-8 kit, was added to the BL side of the Transwell to affect the RAW 264.7 cells. Then, the co-culture system was incubated for 12 h.

### Detection of integrity of monolayer film

When the transepithelial electrical resistance (TEER) values were >400 Ω cm^2^ on the AP side of Transwell plate, the cells were developed to form a dense monolayer (12). The Caco-2 monolayer integrity was monitored at 0, 3, 6, 9, 12, 15, 18, and 21 days through Millicell ERS-2 from Millipore Corporation. The outcomes were denoted as ohm (resistance) × cm^2^ (surface area of the monolayer) and detected 3 times.

On the 21st day, lucifer yellow was indirectly used to prove cell monolayer's integrity (13). The monolayer integrity of the model was evaluated by measuring apparent permeability coefficient (P_*app*_) value of lucifer yellow transported across the monolayer of Caco-2 cells. The P_*app*_ value was expressed as cm/s and calculated according to the following equation.


(3) $$\begin{array}{l} P_{app}=\frac{\frac{dQ}{dt}}{A\times C_{0}} \end{array}$$

where *d*Q/*d*t is the permeation rate of C3G (μg/s) of the BL side, A represents the surface area of the polycarbonate membrane (0.33 cm^2^ for 24-well plates), and C_0_ is the initial concentration of C3G or C3G-NL (0.2 mg/mL).

To evaluate the growth and differentiation characteristics of Caco-2 cells, alkaline phosphatase kit was utilized to measure the enzyme activity on both AP and BL side on the 7th, 14th, and 21st days.

### Co-culture model uptake experiment

#### Time, concentration, temperature

Initially, 200 μL of 10% FBS DMEM containing C3G and C3G-NL (0.2 mg/mL) were incubated on the AP side of co-culture model at equal interval time periods (1, 2, 3, 4, 5, and 6 h), respectively. To explore the effect of C3G and C3G-NL concentration on Caco-2 cell uptake, Caco-2 cells were incubated with various concentrations of C3G and C3G-NL (0.02, 0.04, 0.06, 0.08, 0.1 mg/mL) and the Transwell plate was incubated for 2 h under 37°C. In experiments on the effect of temperature on uptake, 0.2 mg/mL of C3G and C3G-NL were added on the AP side of the co-culture model at different temperatures (4 and 37°C) and incubated for 2 h. Then, the drug-containing culture medium was discarded, the precooled PBS was added to terminate the cell uptake and wash the cell monolayer rapidly three times. Next, 200 μL of cell lysate was added to each well and treated for 5 min. The Caco-2 cells were scraped off into Eppendorf (Ep) tubes with a cell curette and crushed by ultrasound.

#### Uptake of fluorescent probe nanoparticles in Caco-2 cells

To further verify the uptake mechanism, 0.2 mg/mL of Coumarin-6 Nanoliposomes (C6-NL) were added on the AP side of the co-culture model. The models were placed under different conditions (time, concentration, temperature) same as the previous section. After washing the Caco-2 cells (three times) with PBS, the PC membranes were cut with a scalpel and placed on glass slides. Then the nuclear stain DAPI was added to the membranes, and the slides were incubated for 15 min. After washing away the superfluous staining fluid, the distribution of C6-NL in Caco-cells was observed under the fluorescence microscope.

### Co-culture model transport experiment

#### Time, concentration, temperature

Similar to the conditions referred to the previous section, 200 μL of 10% FBS DMEM containing C3G and C3G-NL (0.2 mg/mL) were added on the AP side of co-culture model. Then 100 μL of sample solution was obtained from the BL side at different time periods (1, 2, 3, 4, 5, and 6 h) respectively and 100 μL of 10% FBS DMEM was added. Different concentrations of C3G and C3G-NL (0.02, 0.04, 0.06, 0.08, and 0.1 mg/mL) were added on the AP side of co-culture system. Then 100 μL of sample solution was collected from the BL side 2 h later. In experiments on the effect of temperature on transport, 200 μL of 10% FBS DMEM containing C3G and C3G-NL (0.2 mg/mL) were added on the AP side of co-culture model at different temperatures (4 and 37°C). As mentioned previously, 100 μL of sample solution was obtained from the BL side 2 h later.

#### Endocytosis inhibitors

To investigate the internalization mechanism of the C3G-NL, 100 μL of 10% FBS DMEM containing β-CD (10 mmol/L), chlorpromazine (10 μg/mL), cytochalasin-D (2 μmol/L) were added on the AP side of co-culture model. After 1 h, the added solution was aspirated off. Then, the Caco-2 cells on the AP side of co-culture model were incubated with 200 μL of 10% FBS DMEM containing C3G and C3G-NL (0.2 mg/mL) at 37°C for 2 h. Afterward, 100 μL of sample solution was obtained from the BL side.

#### High-performance liquid chromatography measurement of transported C3G concentration

The C3G concentration in 10% FBS DMEM and Caco-2 cells was detected by high-performance liquid chromatography (HPLC) *via* an Agilent C18 column filled with ZORBAX SB as stationary phase (4.6 × 250 mm, 5 μm; Agilent Technologies Instrument). The test method was slightly modified on the foundations of previous research (10). Solution A (100% acetonitrile) and solution B (1% phosphoric acid in H_2_O) were performed as mobile phases. The solvent gradient was: 0–14 min, 90–76% B; 14–18 min, 76–74% B; 18–22 min, 74–70% B; 22–30 min, 70–0% B; 30–35 min, 0% B; 35–36 min, 0–90% B; 36–45min, 90% B. The flow rate was 0.5 mL/min and peaks were detected at the wavelength of 520 nm. The injection volume was 20 μL and the column temperature was set at 30°C. The external calibration was performed by detecting pure standards with different concentrations.

### Detection of inflammatory cytokines from RAW 264.7 cells

100 μL of 10% FBS DMEM, B-NL, C3G and C3G-NL were added on the AP side of co-culture model. After incubated for 2 h, the concentrations of IL-6, IL-8, IL-1β, and TNF-? were detected in the liquid supernatant on the AP side of co-culture system using the enzyme-linked immunosorbent assay (ELISA) kits with the manufacturer's guidance.

### Statistical analysis

All measurements were performed using Duncan's multiple range test in triplicate, and the original data were presented as mean ± standard error. Statistical analysis was made using the software Origin version 9.0.0. Statistical comparisons were performed by GraphPad Prism software version 9.0. Differences with *P* < 0.05 were regarded as statistic significances.

## Results and discussion

### Characterization of C3G-NL

The particle size, zeta-potential, PDI and EE of nanoliposomes with or without C3G were summarized in Table 1. The mean particle size of C3G-NL was 95.62 ± 0.87 nm resulting in a smaller particle than B-NL (104.29 ± 0.7 nm), which might be due to the application of high-intensity ultrasound propagates sound waves. This produced shear forces causing damage to vesicular structures, followed by the generation of smaller vesicles (14). Also, other relevant studies showed that C3G could interact with phospholipids and reduce the particle size of nanoliposomes due to the modification of the acyl chain order (15, 16). Besides, C3G encapsulated in nanoliposomes affected the dynamic load elastic modulus and membrane fluidity (17).

**Table 1:** Mean particle size, ζ-potential, polydispersity index (PDI) and encapsulation efficiency (EE) of B-NL and C3G-NL.

**Sample**	**Mean particle size (nm)**	**ζ-potential (mV)**	**PDI**	**EE (%)**
B-NL	104.29 ± 0.7	−103.08 ± 1.48	0.283 ± 0.01	–
C3G-NL	95.62 ± 0.87	−108.44 ± 3.94	0.249 ± 0.023	71.70 ± 1.9%

Generally, PDI presented the molecular weight of a polymer from homogeneity to heterogeneity in the range of 0 to 1.0, where homogeneous particles ranged from 0 to 0.3 (18). As shown in Table 1, the PDI of B-NL and C3G-NL were lower than 0.3, implying that the nanoliposomal carriers produced in this study with a narrow particle size distribution was steady and homogenous. When the absolute value of ζ-potential was >30 mV, it indicated that the mixture was transformed into a stable system because high surface charges blocked gathering through the improvement of repulsion between charged granules. Table 1 also illustrated that B-NL and C3G-NL presented a negative surface charge >100 mV, showing that both solution was highly stable.

In addition, the UV spectrophotometry analysis showed the well encapsulation of C3G in liposomal systems with a higher EE reaching 71.70 ± 1.9%. C3G, as a flavonoid with hydrophilic properties, was wrapped into the interior aqueous core of nanoliposomes. According to previous studies, the polysaccharide (chitosan, pectin, *etc*.) as a coating of nanoliposomes, could decrease the membrane fluidity and compound leakage, thus increasing the encapsulation efficiency (19–21).

### Morphology of C3G-NL

**Figure 1:**
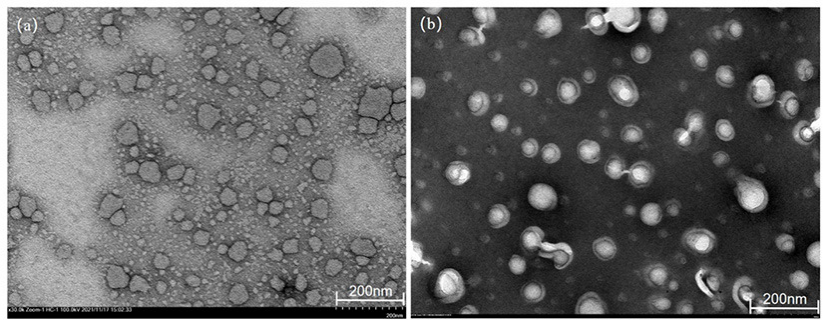
Transmission electron microscopy (TEM) images of **(a)** blank nanoliposomes (B-NL) and **(b)** C3G-loaded nanoliposomes (C3G-NL) prepared.

TEM can detect the size of particles smaller than 0.2 μm, which are called submicroscopic structures or ultrastructures. The morphological features of C3G-NL and B-NL were observed *via* TEM imaging (Figures 1a,b). Both types of nanoliposomes displayed primarily spherical or near-spherical structures and smaller than 100 nm, which was consistent with the above DLS result. Besides, C3G-NL (Figure 1a) had a smaller particle size and smoother surfaces than B-NL. Figure 1b also showed visible small unilamellar vesicles due to the encapsulation of C3G in phospholipid bilayer. The previously published research supported these similar results. Both B-NL and drug-loaded NL were smooth surfaces, spherical in shape, and presented ash or blackish particles (22, 23).

### Infrared spectrum analysis

**Figure 2:**
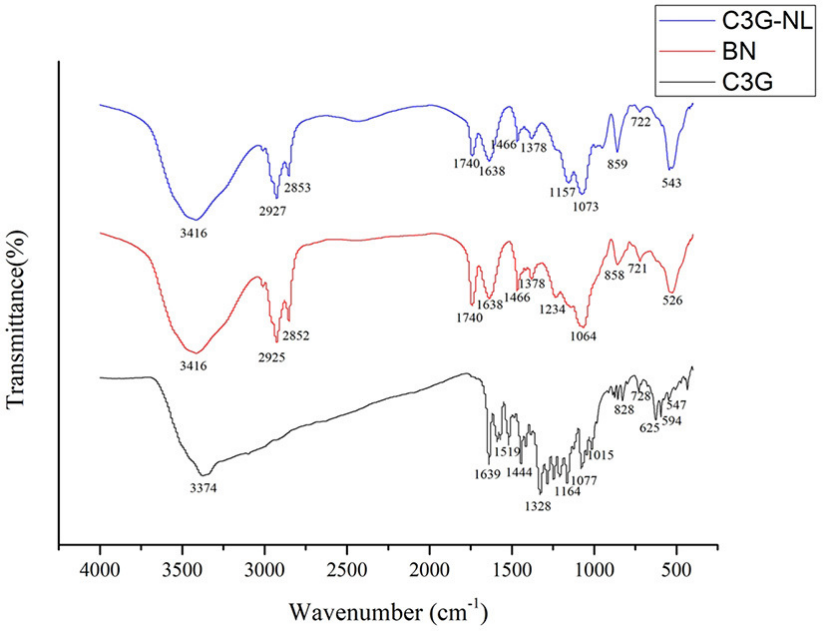
Fourier transform infrared (FTIR) spectra of C3G, B-NL and C3G-NL. The characteristic peaks were labeled.

FTIR analysis was subjected to explore the imperceptible changes in the nanoliposome assemblage profile by analyzing the frequency and bandwidth changes under the mode of vibration (24). Figure 2 showed that the FTIR spectra of C3G, B-NL, and C3G-NL were determined in the wavenumber from 4,000 to 400 cm^−1^. The wave range of 3,374–3,416 cm^−1^ signified the O–H stretch vibrations peaks for CH (25). At the wave numbers of 1,077 cm^−1^, the characteristic peaks showed C–H deformation of the aromatic ring. The stretching vibrations of the C=O in the benzopyran aromatic ring and C=C in aromatic ring could be monitored at the wavenumbers of 1,639 and 1,444 cm^−1^, respectively (26, 27). Moreover, phenols' C–O angular deformations were presented at 1,328 cm^−1^ (26).

In the B-NL sample, the antisymmetric and symmetric stretching of the methylene C–H were situated at the wave of 2,925 and 2,852 cm^−1^, respectively (28). And B-NL indicated other specific bands of lipid bilayers located at 1,234 and 1,740 cm^−1^, referring to stretch vibrations of the P=O and the C=O in ester groups, respectively (29). The intensity of peaks of C3G-NL differed from those of B-NL slightly in the FTIR spectrum. The changes from 1,234 to 1,157 cm^−1^ corresponded to the stretching vibration mode of P=O bond, illustrating that the configuration and composition of phospholipid bilayer in C3G-NL were influenced by the existence of C3G (30). The ${\text{PO}}_{2}^{-}$ symmetric and asymmetric stretching vibration of phospholipid were monitored at the wavenumber of 1,378 and 1,073 cm^−1^, respectively (31). The specific bands of C3G were not found obviously in C3G-NL, showing that the C3G would be encapsulated into the nanoliposomes well.

### Viability of C3G and C3G-NL by Caco-2/RAW 264.7 co-culture systems

**Figure 3:**
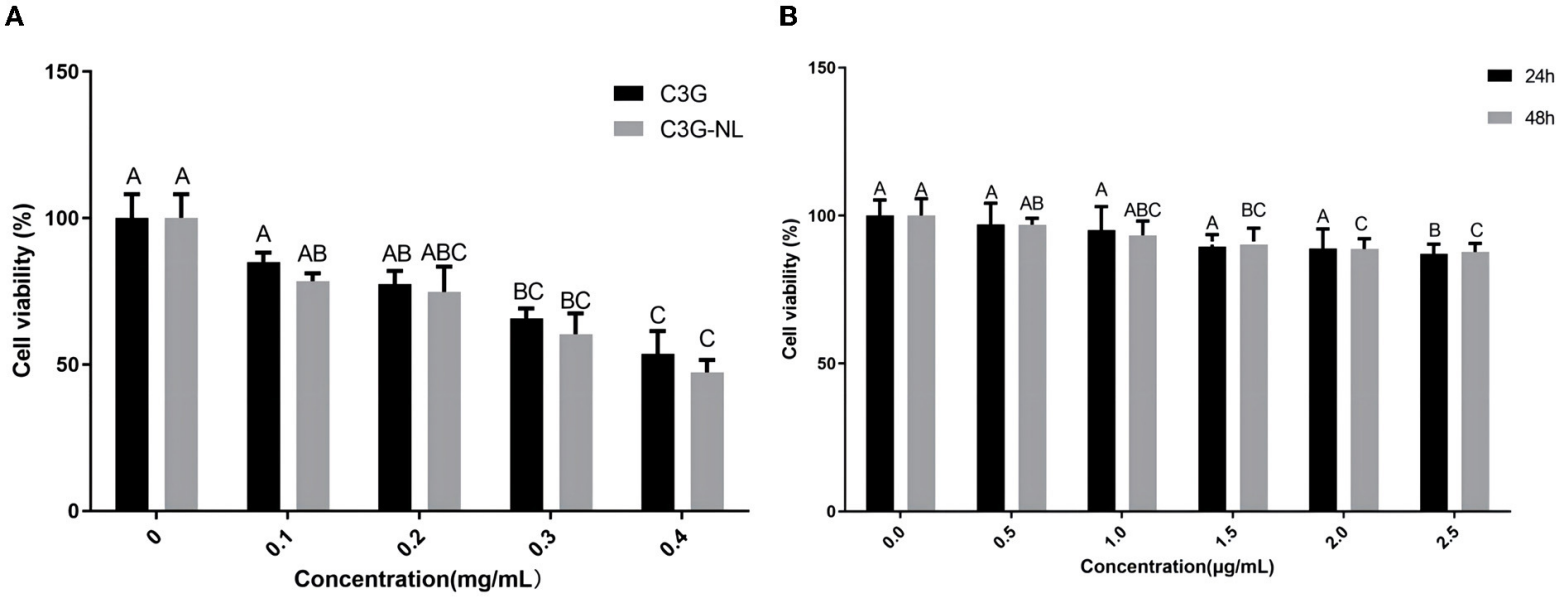
Cell viability. **(A)** Cell viability of Caco-2 cells treated with different concentrations of C3G or C3G-NL (*n* = 6 times per group). **(B)** Cell viability of RAW 264.7 cells treated with various concentrations of LPS (*n* = 6 times per group). Different uppercase letters represent significant differences between groups (*P* < 0.05).

As depicted in Figure 3A, both C3G and C3G-NL showed concentration-dependent inhibitory effects on Caco-2 cell viability. Compared to the control group, cell viability was not obviously fluctuated by C3G in the range of 0–0.2 mg/mL, which is consistent with the previous research (10). Caco-2 cells incubated with C3G-NL presented lower viability than those incubated with C3G under the same concentration. As is known to all, C3G could inhibit the proliferation of cancer cells and the presence of lecithin increases the release efficiency of C3G (2, 4). Furthermore, we found the survival rate of RAW 264.7 cells treated with LPS was more than 70% in the range of 0–2.5 μg/mL, indicating no significant differences of cytotoxicity of the LPS among all the groups (Figure 3B).

### Detection of integrity of monolayer film

**Figure 4:**
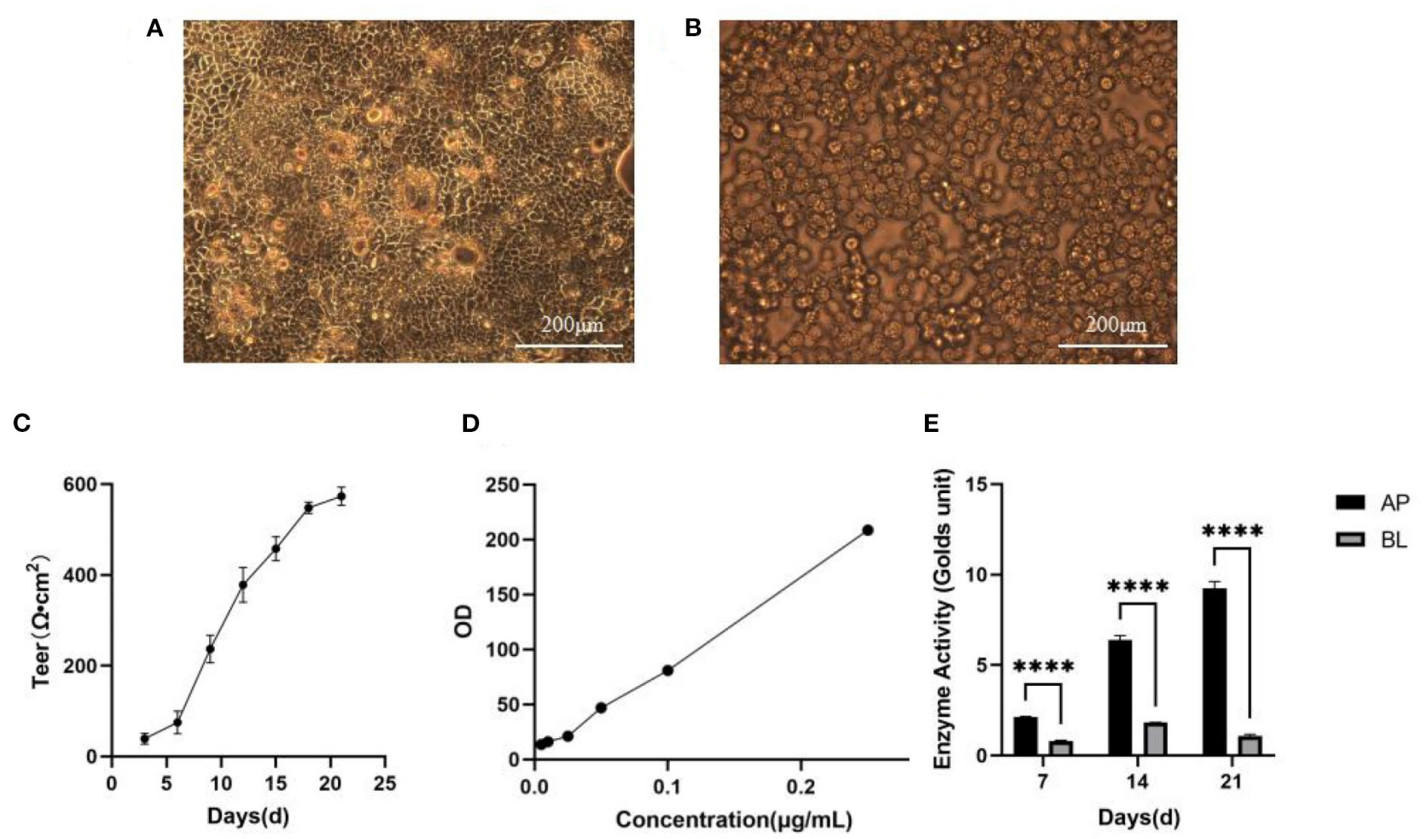
Establishment of Caco-2 cell model. **(A)** Morphology of Caco-2 cells observed in 21th day. Scale bar = 200 μm. **(B)** Morphology of RAW264.7 cells observed in 2th day. Scale bar = 200 μm. **(C)** TEER of Caco-2 cells with time (*n* = 3 times per day). **(D)** The standard curve of lucifer yellow. **(E)** Alkaline phosphatase activities. ****p < 0.0001 compared with BL side (*n* = 3 times per group).

Figure 4A illustrated that the monolayer membrane of Caco-2 cells had good integrity without gaps when cultured for 21 days. Also, when the RAW 264.7 cells were incubated in the BL side on the 3rd day, most were round or oval, small and bright (Figure 4B). The integrity of Caco-2 cell monolayers could be directly represented *via* transepithelial electrical resistance (TEER). As shown in Figure 4C, the TEER value reached 556 Ω·cm^2^ on the 21st day, implicating that the Caco-2 cells monolayer was dense and intact so that the system could be utilized to the next experimental procedure. Besides, the monolayer integrity was indirectly confirmed by lucifer yellow transport assay (Figure 4D), the permeability of lucifer yellow with Caco-2 cells was validated as 3.03 × 10^−8^ cm/s *via* the equation of linear regression of lucifer yellow absorbance [*y* = 803.6*x* + 5.623 (*R*^2^ = 0.9974)]. The results further proved that Caco-2 cells had experienced complete differentiation and the cell monolayer achieved morphological integrity after 21 days. Moreover, the alkaline phosphatase activity of both sides could be displayed in Figure 4E, it was clear that the activity on the AP side was ~4 times higher than that on the BL side on the 21st day. Therefore, this model processed the suitable function and condition for following transport and uptake experiments (13).

### Uptake of C3G nanoliposomes by Caco-2/RAW 264.7 co-culture model

**Figure 5:**
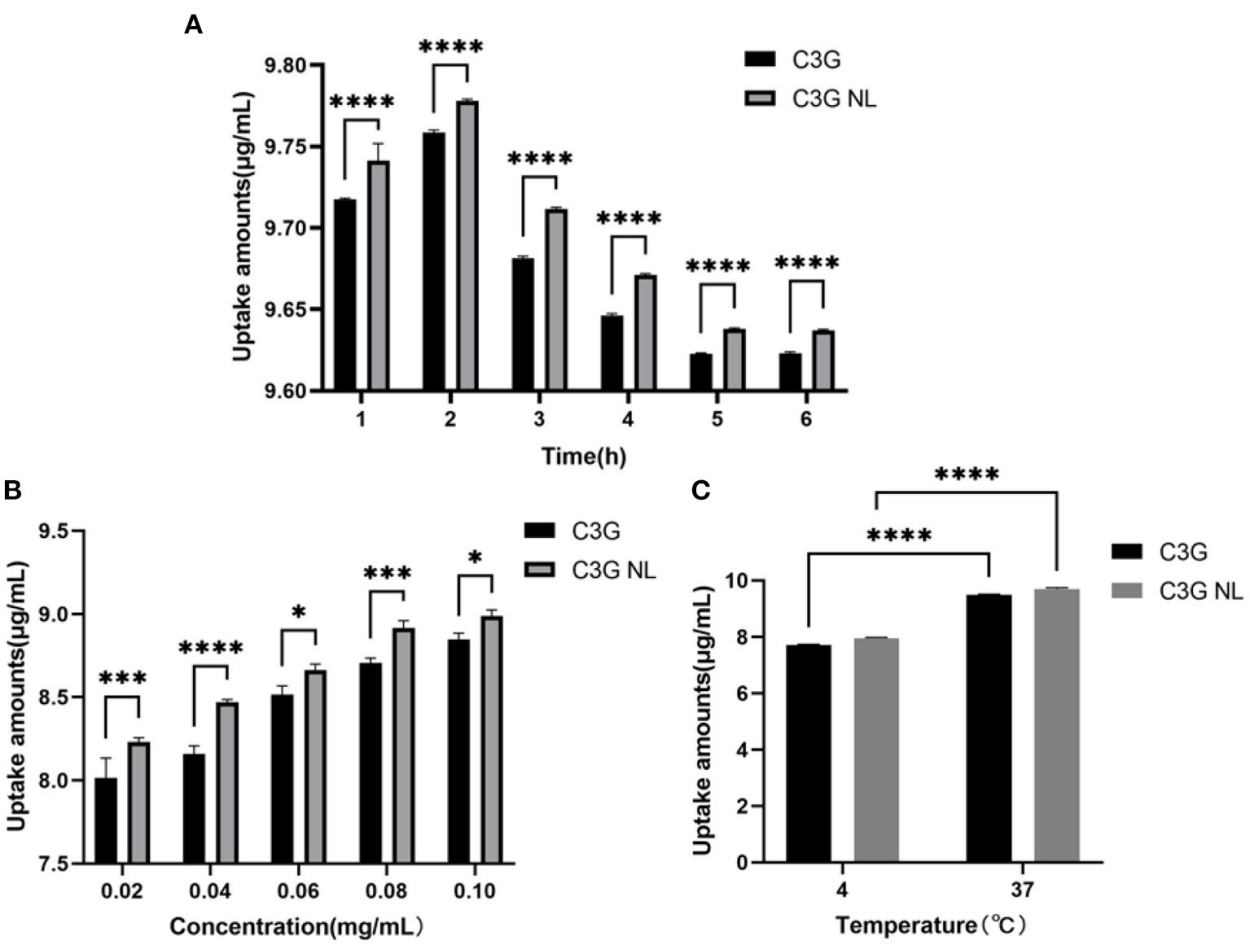
The uptake of C3G and C3G-NL with different concentrations (0.02–0.10 mg/mL) of cells during 6 h. **(A)** The effect on the uptake of C3G and C3G-NL (0.2 mg/mL) at different times. ****p < 0.0001 compared with C3G group (*n* = 3 times per group). **(B)** The effect of concentration on uptake of C3G and C3G-NL. *, ***, and **** represent *P* < 0.05, 0.001, and 0.0001, respectively, compared with the C3G group (*n* = 3 times per group). **(C)** The effect of temperature on uptake of C3G and C3G-NL. ****p < 0.0001 compared with 4°C group (*n* = 3 times per group).

To explore the bioavailability of free-C3G and C3G-loaded nanoliposomes on the cellular intestinal inflammatory model, C3G and C3G-NL were treated in the co-culture model at different times, concentrations and temperatures. Then C3G concentrations in Caco-2 cells were analyzed and compared quantificationally through ultrasonic dispersion. Passive diffusion is the primary uptake mechanism of C3G by colon cancer cells, and many active transporters are involved depending on structure feature of cells. (32). The uptake of C3G and C3G-NL was illustrated using a moderate and available concentration (0.2 mg/mL) by co-culture models. Under the condition of 37°C, the uptake of C3G and C3G-NL in Caco-2 cells increased firstly, which peaked at 2 h and then decreased until 6 h (Figure 5A). The results showed that the suitable time on the intake amounts of C3G and C3G-NL in Caco-2 cells was 2 h, which would be the “timing” for the subsequent uptake experiments. Compared with the free-C3G group, C3G-NL significantly improved cellular uptake of the ACNs. This also accorded with our earlier observations, which showed that the nanoliposomes with membrane-like structure might enhance the capacity for cellular uptake (33, 34).

As illustrated in Figure 5B, the uptake rate of C3G and C3G-NL by Caco-2 monolayer cells on the AP side enhanced as the concentration increased and seemed not to reach saturation under the condition of 37°C and 2 h. Figure 5C also indicated that the intake of C3G and C3G-NL at 4°C was significantly reduced compared with those at 37°C, as low temperature affects the fluidity of cell membranes. These results showed that the cellular uptake was concentration-dependent and energy-dependent. As mentioned in the literature review, nanoliposomes could enter cells with drugs through endocytosis such as clathrin-dependent endocytosis, caveolae-dependent endocytosis, macrocytosis and fusion, *etc* (35). Combined with previous literature (36), it was speculated that C3G-NL might follow a combination of endocytosis and concentration-dependent passive diffusion. The cell category, configuration, surface charge, and size of the nano-particles would influence the mechanism of the nanoliposome internalized into the cells (37).

### The uptake of coumarin-6 (C6)-loaded lipid nanoparticles in Caco-2/RAW 264.7 co-culture model

**Figure 6:**
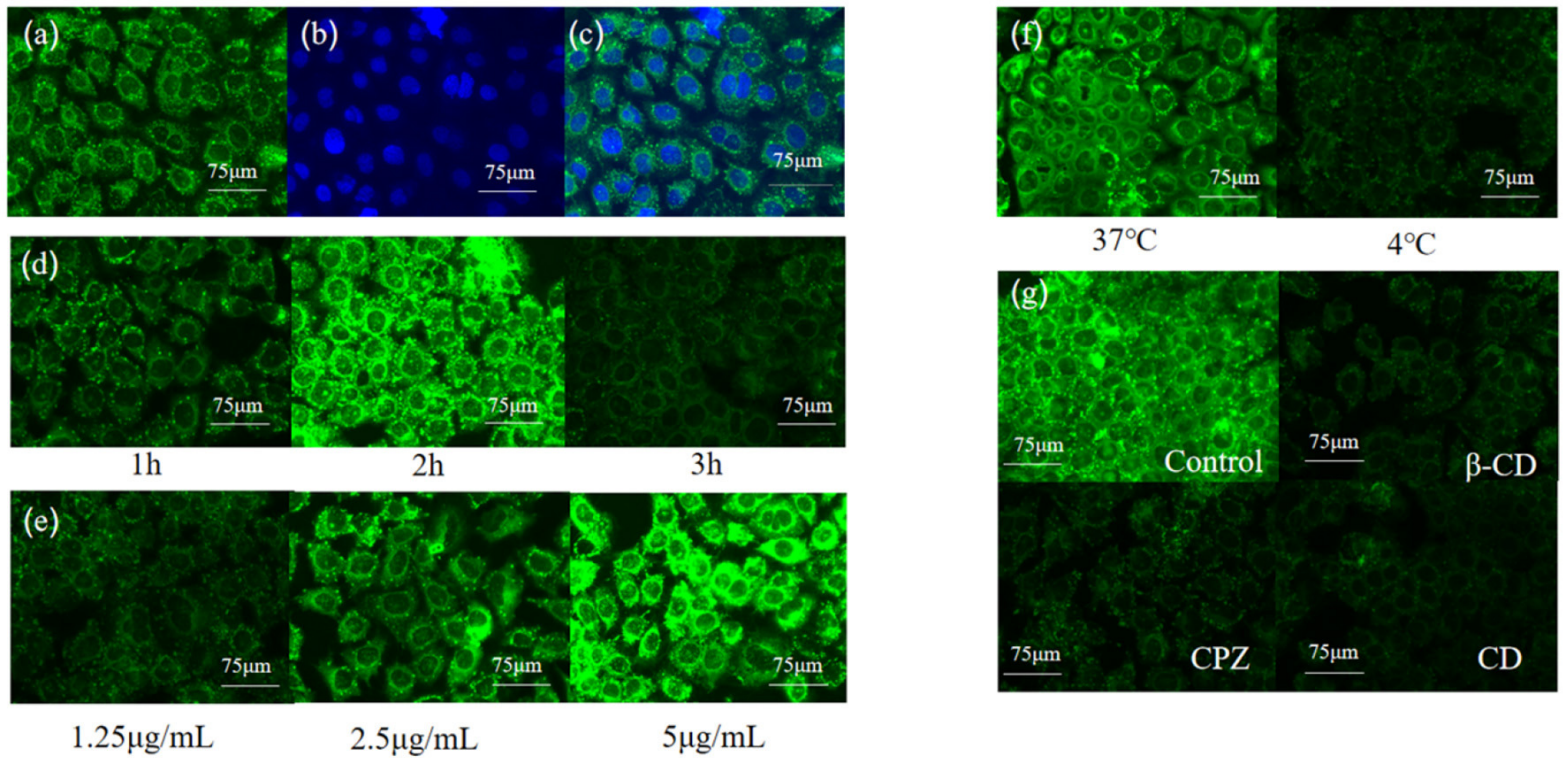
Relationship between uptake amounts of Coumarin-6 solid lipid nanoparticles and time, concentration, temperature and endocytic inhibitors (bar: 75 μm) **(a)** Coumarin-6; **(b)** DPAI; **(c)** merge; **(d)** time; **(e)** concentration; **(f)** temperature; **(g)** endocytic inhibitors.

To illustrate the uptake mechanism of C3G-NL to Caco-2/RAW 264.7 co-culture system, we encapsulated the hydrophilic dye Coumarin-6 (C6) in liposomal systems. Green fluorescence was presented in the group of C6-marked nanoliposomes (Figure 6a), and the nucleus exhibited blue as dyed *via* DAPI (Figure 6b). Some cell nucleus performed deformity (Figure 6c) due to the encapsulation of nanoliposomes, which demonstrated that C6-labeled nanoliposomes might have been internalized into the nucleus of Caco-2 cells (11). Next, we determined the cellular uptake of C6-labeled nanoliposomes ingested in Caco-2 cells from AP side of co-culture model for 1, 2 and 3 h through fluorescence microscopy. The results showed the fluorescence intensity was the strongest at 2 h and the Caco-2 cells nucleus was surrounded by the green particles (Figure 6d). The results of treating the Caco-2 cells with different concentrations of C6 liposomes (1.25, 2.5, and 5 μg/mL) and temperature (4, 37°C) are shown in Figures 6e,f, indicating the significant dose-dependent and energy-dependent effect. Similarly, it was reported that the uptake efficiency of baicalin (a type of flavonoids) nanoliposomes in Caco-2 cells was fastest from 0 to 0.5 h, next the absorption rate gradually reduced with the increase of time (38). Compared to the control group, the fluorescence intensity of cells decreased obviously in the β-CD, CPZ and CD groups (Figure 6g). The results further proved that the cellular uptake mechanism of nanoliposomes on Caco-2/RAW 264.7 co-culture model was concentration-dependent and energy-dependent. Moreover, endocytosis was proved to participate in the uptake of nanoliposomes, which was in line with this study's. In contrast, quercetin, a flavonoid structurally similar to anthocyanins, presented no significant difference in C3G-NL absorption, which illustrated that the uptake mechanism of the C3G-NL did not depend on clathrin- and caveolae-independent pathways (36). Thus, the uptake mechanism of C3G should be further discussed.

### Transport of C3G nanoliposomes by Caco-2/RAW 264.7 co-culture system

**Figure 7:**
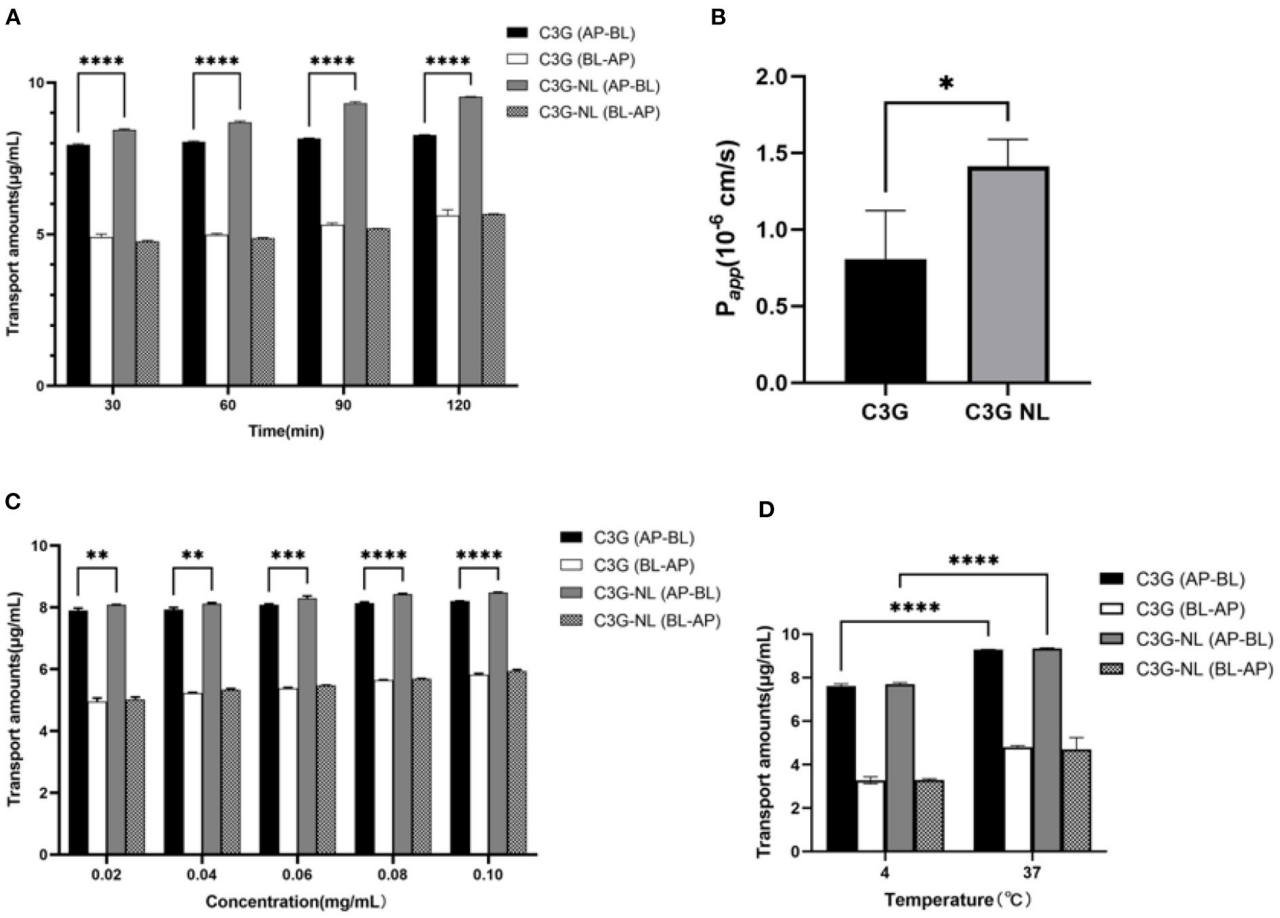
Effects of different time, concentrations and temperatures on the transport of C3G and C3G-NL. **(A)** The effects of AP-BL and BL-AP on the transport of C3G and C3G-NL (0.2 mg/mL) at different times (*n* = 3 times per group). **(B)** P_*app*_ values of free- and nano-C3G (*n* = 3 times per group). **(C)** The transport of C3G and C3G-NL with different concentrations (0.02–0.1 mg/mL). *, **, ***, and **** represent *P* < 0.05, 0.01, 0.001, and 0.0001, respectively, compared with the C3G group (*n* = 3 times per group). **(D)** Effects of different temperatures on C3G and C3G-NL transport. ****p < 0.0001 compared with 4°C group (*n* = 3 times per group).

In experiments on the effect of time on transport, the C3G and C3G-NL transport amounts on both sides of co-culture model gradually enhanced. Then the transport amount reached the maximum at 120 min but tended to be unsaturated. Intriguingly, 9.532 ± 0.01 mg/mL C3G-NL was detected in the BL side after 120 min and the transport amounts of C3G-NL was 1.13-fold that of C3G (*P* < 0.0001), which demonstrated that nano-particles could significantly enhance the permeability of C3G (Figure 7A). Following the present results, the amounts of C3G transported across the Caco-2 monolayers enhanced linearly with the extension of ingested time (36). As shown in Figure 7B, the P_*app*_ value of pure C3G was proved to be (0.81 ± 0.32) × 10^−6^ cm/s which was below 1 × 10^−6^ cm/s. This cutoff illustrated the cell monolayer processed a high permeability (39). The P_*app*_ value of C3G-NL was 1.75-fold that of C3G (*P* < 0.05), which were in accord with recent studies indicating that ferritin nanocage might increase the release rate of C3G from 2.51 to 2.71 mg transported across the cell monolayers through adaptor complex 2-dependent pathway (40).

Under the condition of 37°C, it was clearly observed that the transport volume of C3G and C3G-NL in the co-culture system gradually enhanced as concentrations increased with the same upward trend as time. At the same concentration, the transport amount of C3G NL group from AP side to BL side was significantly higher (*P* < 0.01) than that of C3G group from AP side to BL side (Figure 7C). The maximum C3G-NL transport amount on the AP-BL side was 8.478 ± 0.013 mg/mL, and the maximum C3G transport amount on the AP-BL side was 8.206 ± 0.005 mg/mL. Moreover, the maximum C3G-NL transport amount on the BL-AP side was 5.942 ± 0.05 mg/mL, and the AP-BL side was significantly higher than the BL-AP side. Moreover, we explored the effect of temperature on the transport of C3G and C3G-NL under the condition of 37 and 4°C. Compared to 37°C, the transshipment amount in co-culture system decreased at 4°C significantly, suggesting that low temperature has a negative effect on transport ability of cells (Figure 7D). This result is consistent with the conclusion in the previous uptake experiments. Besides, it was illustrated that the uptake volume of nano-Cur in zein–sodium caseinate nanoparticles was about twofold more than that of the free Cur. Nanocarriers, which were proved the nano-particles could directly enter cells *via* endocytosis (41). Next, relevant experiments are exhibited to reveal whether the transport mechanism of C3G-NL was endocytosis.

### The impact of endocytosis inhibitors on C3G-NL transport

**Figure 8:**
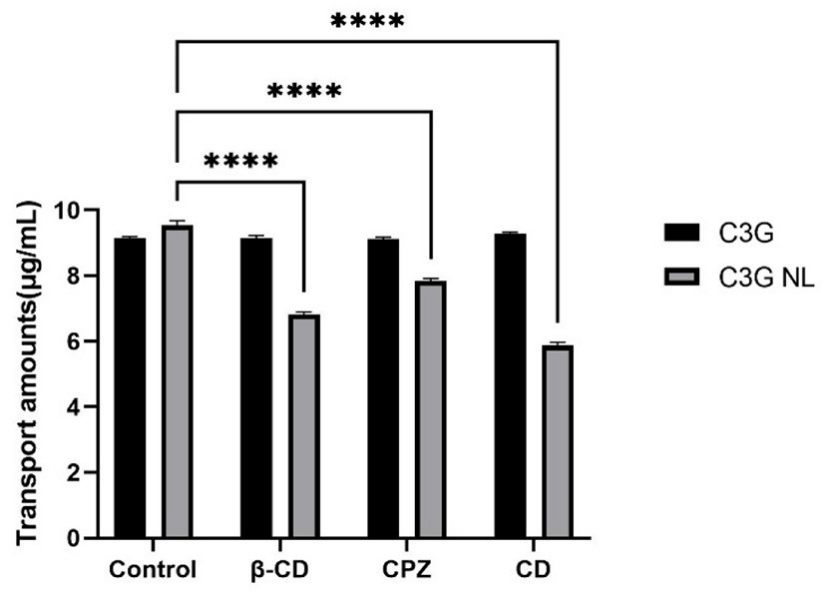
Effects of various endocytosis inhibitors on the cellular transport of C3G.and C3G-NL. **** represent *P* < 0.0001, compared with the control (*n* = 3 times per group).

As mentioned above, the transport volume of C3G-NL in the co-culture model from the AP side to the BL side decreased significantly when incubated at 4°C (*P* < 0.05). This prevented production of ATP in cells, implicating that the C3G internalization depends on energy (42). The endocytosis of nanoliposomes by enterocyte was active and energy-dependent, divided into the clathrin-related route, the caveolae-related route, macropinocytosis, and clathrin- and caveolae-independent routes (43). Figure 8 showed water soluble methyl β-cyclodextrin (β-CD), chlorpromazine (CPZ) and cytochalasin D (CD) caused diverse effects on the transport rate of C3G nanoliposome. After the C3G and C3G-NL administration in the apical side, Caco-2's transport of C3G was not significantly changed after incubated with β-CD, CPZ and CD, compared to the blank group, while the transport amounts of C3G-NL were significantly reduced.

β-CD could combine with cholesterol in the cell membrane, thus causing the damage to the integrity of caveolae (44). The transport amounts of C3G-NL from AP side to BL side reduced significantly (*P* < 0.0001) with the addition of β-CD, which exhibited that the caveolae-mediated pathway was also involved. CPZ could inhibit the function of clathrin on the plasma membrane, thus suppressing clathrin-related endocytosis (45). Compared to control group, CPZ experimental group caused a one-fifth decrease in the transport amount of C3G-NL (*P* < 0.01). Thus, it was proved the clathrin-related pathway participated in the endocytosis of the C3G-NL. Nanoliposomes mainly enter cells through the clathrin-related pathway, which occurred commonly to nonionic micelles, anionic protein nanoparticles, and cationic chitosan nanoparticles (4, 46). Different from clathrin- and caveolae-mediated routes, extracellular nanoparticles performing the cells macropinocytosis are occurred in cell membrane, producing macropinosomes with a diameter larger than 1 mm (47). CD, inhibiting actin filament-related absorption, restrained the transport amounts of C3G-NL at ~38.51% (*P* < 0.0001), which illustrated the macropinocytosis route controlled the endocytosis of the C3G-NL. Prior studies have noted that granules with a diameter of about 100 nm could enter cells *via* the macropinocytosis pathway (36), and the mean size of the C3G-NL in this trial was 95.62 nm (Table 1). To summarize, C3G-NL enter cells by both the clathrin- and caveolaerelated routes and the macropinocytosis pathway.

In summary, the transcytosis of the C3G-NL occurred mainly *via* the clathrin-related route and the macropinocytosis pathway. Similarly, a related report subject to SPI nanoparticles showed that the endocytosis pathway of them depended on size of the particles. Moreover, the transport of 30 nm nanoparticles could be mediated through clathrin-related pathway and macropinocytosis, while the transport of 100 nm nanoparticles could be related to micropinocytosis (46).

### Inhibitory effects of C3G and C3G-NL on cytokine secretion

Chronic inflammation is related to metabolic disturbance, neurodegenerations, and other chronic diseases such as premature senility, muscular fatigue, and myocarditis. As mentioned in the literature review, various gut cytokines, growth factors, and transcription factors could be generated in local tissues by endotoxins or dietary structures (8). The steady state and tissue functions could be disrupted by changes of this complex network, which would lead to the occurrence of systemic inflammatory syndrome. The Caco-2/RAW 264.7 cells co-culture model has been utilized to solve the immune–metabolic crosstalk by incubating Caco-2 cells in the AP side and RAW 264.7 cells in the BL side (48). We will explore the mechanism of C3G suppressing intestinal inflammation and relevant endothelial inflammation in a dynamical co-culture system.

**Figure 9:**
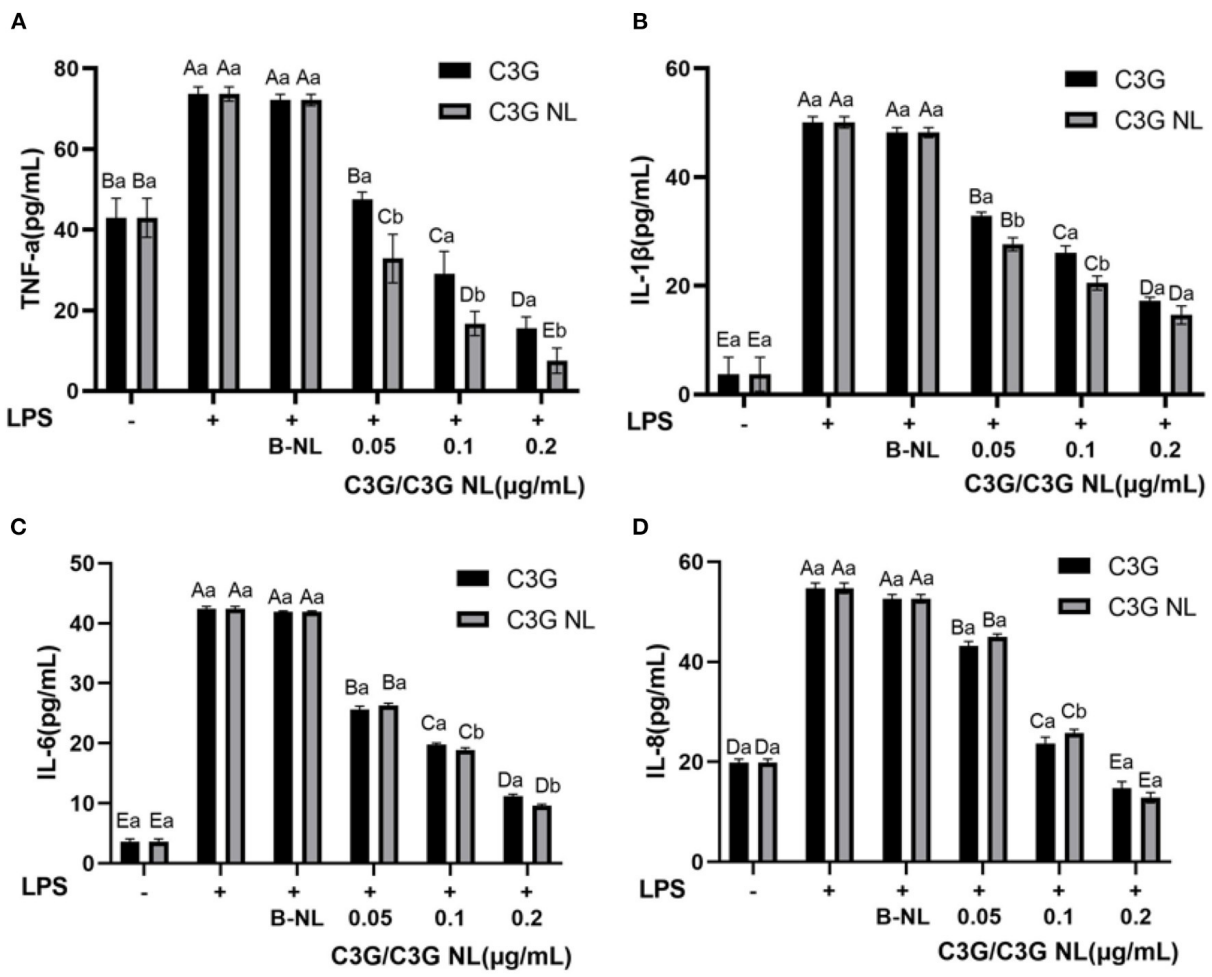
The profile of inflammatory cytokines in co-culture system treated with LPS for 48 h. **(A)** TNF-α; **(B)** IL-1β; **(C)** IL-6; **(D)** IL-8. LPS, lipopolysaccharide. Different uppercase and lowercase letters represent significant differences within and between groups, respectively (*P* < 0.05, *n* = 3 times per group).

A previous study has demonstrated that, C3G, as one of the primary anthocyanins in the colorful fruit, presented significant anti-inflammatory activity (49). Compared to the control group, the production level of TNF-α, IL-1β, IL-6, IL-8 in the LPS group presented 1.7-, 13.5-, 11.8-, and 2.8-fold increase (Figure 9). In contrast, C3G and C3G-NL treatment groups reduced significantly in the concentrations of TNF-α, IL-1β, IL-6, IL-8 compared to the LPS group (*P* < 0.05). When treated at the highest concentration of C3G and C3G-NL, the production of TNF-α, IL-1β, IL-6 and IL-8 was significantly reduced by about 79, 66, 74, 73, 90, 71, 77, and 77%, respectively, compared to the LPS group. In the highest concentration, TNF-α, IL-1β, and IL-8 were dwindled significantly between C3G and C3G-NL group except for IL-6. This study accorded with the earlier research, indicating that C3G from berries had a significant anti-inflammatory effect (48). The treatment of C3G could promote to reduce inflammatory activity by a co-culture model consisting of Caco-2 cells and RAW 264.7 cells simulating intestinal inflammation.

The nuclear factor κB (NF-κB) and the mitogen-activated protein kinase (MAPK) pathways were two mainly pro-inflammatory signaling pathways to regulate the cellular inflammatory reactions, such as the generation of inflammatory cytokines (50). It had been previously proposed that the increase of nuclear NF-kB accumulation induced by TNF-α and the expression of IL-6 was significantly inhibited by C3G pretreatment (51). As this study illustrated, C3G inhibited the inflammatory reaction induced through LPS, as well as restrained the production of TNF-α, IL-1β, IL-6, and IL-8 in Caco-2/RAW 264.7 cells co-cultured system. However, the present study has several limitations. On the one hand, we could not confirm which specific metabolites and derivatives are responsible for the anti-inflammatory activity. On the other hand, the anti-inflammatory mechanism of C3G is associated with NF-κB, such as TJs, occludin, claudins, JAMs, phosphorylation and JNK and IκB-α, and other specific inflammatory pathways were not investigated (52).

## Conclusion

In this work, we successfully developed nanoliposomes with a high encapsulation rate, small diameter, suitable ζ-potential value, and low polydispersity index using a thin-layer dispersion method combined with ultrasonication. Compared with free-C3G, nanoliposomes improved the uptake and transport of C3G and enhanced the anti-inflammatory activity of C3G. Endocytosis and macropinocytosis are the main routes for C3G uptake in an intestinal inflammation model. C3G and C3G-NL could restrain intestinal inflammation by inhibiting the production of inflammatory cytokines, such as TNF-α, IL-1β, IL-6, and IL-8. In future research, we will explore the expression of uptake-associated factors and proteins and specific signaling pathways of C3G in attenuating intestinal inflammation.

## Data availability statement

The original contributions presented in the study are included in the article/Supplementary material, further inquiries can be directed to the corresponding authors.

## Author contributions

MY: conceptualization, methodology, writing—original draft, writing—review, and editing. XLu and WZ: preparation of figure, reviewing, editing, and visualization. JX: performance of statistical data analysis. XLiu: review and editing. RG and HZ: conceptualization and supervision. All authors contributed to the article and approved the submitted version.

## Funding

The present research was supported by National Natural Science Foundation of China (No. 32172202) and Key Technology Research and Development Program of Natural Science Foundation of Zhejiang Province (No. 2021C04032).

## Conflict of interest

The authors declare that the research was conducted in the absence of any commercial or financial relationships that could be construed as a potential conflict of interest.

## Publisher's note

All claims expressed in this article are solely those of the authors and do not necessarily represent those of their affiliated organizations, or those of the publisher, the editors and the reviewers. Any product that may be evaluated in this article, or claim that may be made by its manufacturer, is not guaranteed or endorsed by the publisher.
