# Supplementary material for: Cellular uptake, transport mechanism and anti-inflammatory effect of cyanidin-3-glucoside nanoliposomes in Caco-2/RAW 264.7 co-culture model
Source: Front Nutr. 2022 Sep 26;9:995391. doi: 10.3389/fnut.2022.995391 (PMC9549275; doi:10.3389/fnut.2022.995391)
Supplement: Supplementary file 2 [file Table_2.DOCX]

**
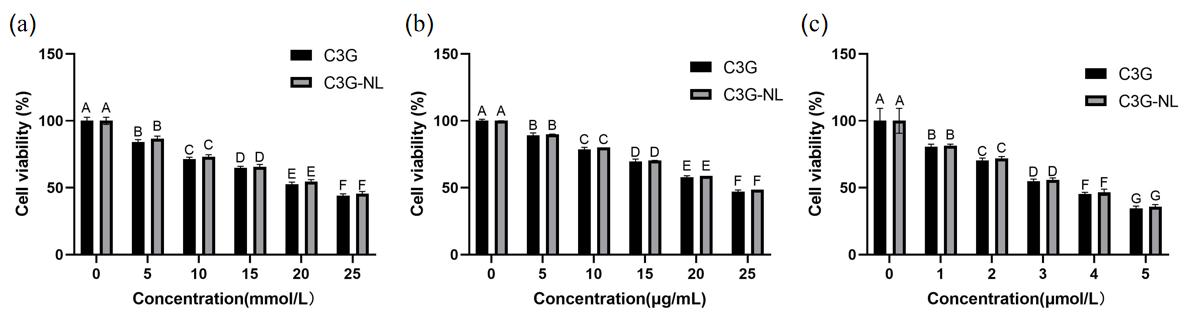
**

**FIGURE S1 Cell viability of Caco-2 cells with inhibitors.** Cell viability of Caco-2 cells treated with different concentrations of (a) β-CD; (b) CPZ; (c) CD. Different uppercase letters represent significant differences between groups (P < 0.05).
